# Plant Species and Heavy Metals Affect Biodiversity of Microbial Communities Associated With Metal-Tolerant Plants in Metalliferous Soils

**DOI:** 10.3389/fmicb.2018.01425

**Published:** 2018-07-16

**Authors:** Sławomir Borymski, Mariusz Cycoń, Manfred Beckmann, Luis A. J. Mur, Zofia Piotrowska-Seget

**Affiliations:** ^1^Department of Microbiology, Faculty of Biology and Environmental Protection, University of Silesia, Katowice, Poland; ^2^Department of Microbiology and Virology, School of Pharmacy with the Division of Laboratory Medicine, Medical University of Silesia, Sosnowiec, Poland; ^3^Institute of Biological, Environmental and Rural Sciences, Aberystwyth University, Aberystwyth, United Kingdom

**Keywords:** heavy metals, rhizosphere biodiversity, BIOLOG-CLPP, PLFA, DGGE, HPLC-MS, metallophytes

## Abstract

We here assess the biodiversity of the rhizosphere microbial communities of metal-tolerant plant species *Arabidopsis arenosa, Arabidopsis halleri, Deschampsia caespitosa*, and *Silene vulgaris* when growing on various heavy metal polluted sites. Our broad-spectrum analyses included counts for total and metal-tolerant culturable bacteria, assessments of microbial community structure by phospholipid fatty acid (PLFA) profiling and community-level analysis based on BIOLOG-CLPP to indicate functional diversity. The genetic-biochemical diversity was also measured by denaturing gradient gel electrophoresis (PCR-DGGE) and metabolomic analysis (HPLC-MS). Different rhizospheres showed distinctive profiles of microbial traits, which also differed significantly from bulk soil, indicating an influence from sampling site as well as plant species. However, total bacterial counts and PCR-DGGE profiles were most affected by the plants, whereas sampling site-connected variability was predominant for the PLFA profiles and an interaction of both factors for BIOLOG-CLPP. Correlations were also observed between pH, total and bioavailable Cd or Zn and measured microbial traits. Thus, both plant species and heavy-metals were shown to be major determinants of microbial community structure and function.

## Introduction

Heavy metals pose a serious threat to soil organisms and the entire ecosystem when they occur at excessive amounts. Despite the high toxicity of metals to plants, some plants have developed mechanisms that enable them to survive in environments with elevated metal levels. These plants include obligate metallophytes (true metallophytes), which are often endemic to their native metalliferous sites and facultative metallophytes (pseudometallophytes), which can grow on both, polluted and not-polluted soils (Lucassen et al., 2009; Baker et al., 2010; van der Ent et al., 2013). Metalliferous soils often represent sandy, nutrient-poor and dry substrates where plant coverage is low and patchy. Since the early work of Lorenz Hiltner, who coined a term “rhizosphere” in 1904, it is known that plants create a specific niche where microbial growth and activity become intensified (Hartmann et al., 2008). This rhizosphere effect is thought to arise from the deposition of various organic compounds from plants into the soil (Prashar et al., 2014). Plants exudate a variety of small and high molecular weight organic as well as inorganic compounds that enrich rhizosphere in nutrients that attract and stimulate soil microbial communities. For this reason, rhizosphere microbial communities tend to have higher microbial counts and generally show higher activity than those occurring in bulk soil. It has been estimated that between 10 and 40% of carbon assimilated by plants can be released into rhizosphere in various compounds, like amino acids, organic acids, phenolic compounds, proteins and polysaccharides (Bais et al., 2006). Plant-related compounds released to soil environment may also act as messengers that initiate interactions between roots and a wide range of soil organisms (Perrine-Walker et al., 2011).

It is believed, that plants select the bacteria that inhabit the soil around their roots. Therefore, the metallophyte rhizosphere may provide a nutrient-rich microenvironment that allows certain microbial communities to thrive or aid in the conferring metal tolerance. In line with this, different patterns of exudate composition have been observed for individual plant species and even individual genotypes (García-Villaraco Velasco et al., 2009). The rhizosphere of metallophytes typically consists of heavy metal tolerant bacteria, and thereby acts as a reservoir for specialized metal-tolerant microorganisms (Dechamps et al., 2005; Alford et al., 2010). Additionally, some metal-tolerant bacteria, especially plant growth-promoting bacteria, are known to enhance the phytoremediation of soils contaminated with heavy metals (Lasat et al., 2001; Abou-Shanab et al., 2003). Thus, both plants and microorganisms interact with themselves and surrounding soil environment to affect soil quality and function. Such functions must be centered on maintaining nutrient cycling and element turnover, which corresponds directly to energy transfer, biomass production and organic matter decay (Yan et al., 2008; Carrasco et al., 2010). In heavy metal contaminated soils, like mine tailings, which are often nutrient-poor, maintenance of these processes is a function of the metallophyte plants and microorganisms accompanying them (Zhang et al., 2012). Given such observations, studies on microbial community biodiversity linked with metal-tolerant plants and plant-soil-microbe interactions are of great importance for a better understanding of soil function as impacted by metallic stress. Moreover, understanding the relationship between the bacteria surrounding the roots of hyperaccumulators and pseudometallophytes is a key first step toward the extensive use of these plants in phytoremediation (Whiting et al., 2001; Abou-Shanab et al., 2006). The aim of this study was to provide a detailed structural and functional analyses of arrangement of plant-soil-microbe axes under condition of heavy metal pollution. We show that both plant species and heavy-metal contamination are major determinants of microbial community structure and function.

## Materials and methods

### Sample collection for the experiment

Rhizosphere soil samples of *Arabidopsis arenosa* (AA), *Arabidopsis halleri* (AH), *Deschampsia caespitosa* (DC) and *Silene vulgaris* (SV) and bulk soils (BK) were collected at the three separate heavy metal polluted areas in Poland, on June 16th 2012. The sampling time has been chosen based on the flowering periods of plants of interest. All plants were in their flowering stage during sample collection. The sampling sites consisted of a 50-year old zinc smelter waste heap in Piekary Slaskie-Brzozowice (site P - 50°22′02.8″N 18°58′19.2″E), and two sites located near the zinc works: 2 km to the north-east (site N - 50°30′48.2″N 18°56′47.1″E) and 2 km to the west (site W - 50°30′20.7″N 18°53′37.1″E) of a zinc works in Miasteczko Slaskie, Upper Silesia. All of the samples were collected during a single day. Blocks that included the root systems of individual plant species were excavated and shaken in order to remove loosely attached bulk soil. The blocks were collected randomly in triplicates within an area of 25 m^2^ for each plant species per site. Any soil that adhered to the root systems was subsequently brushed off and considered to be the rhizosphere. Prior to analysis both the bulk soils and rhizospheres were sieved through 2 mm mesh in order to remove any gravel and plant debris.

### Soil characteristics

Physico-chemical analyses involved the determination of the soil moisture, organic matter (SOM) content, pH, conductivity and the concentrations of Zn, Cd, Cu, and Ni ions. SOM was determined using a loss-on-ignition (LOI) method (EN 13039:2000). Moisture content was obtained by drying at 105°C and weighing in order to estimate mass loss (ISO 16586:2003). The measurement of the total heavy metal concentrations was carried out with use of inductively coupled plasma optical emission spectrometry (ICP-OES) (PN-EN 12457-2:2006). The bioavailable fraction of heavy metals was also assessed. Briefly, five g of dried soil samples were suspended in 50 mL of 0.01 M CaCl_2_ solution and shaken in an orbital shaker for 2 h at 120 rpm to measure the bioavailable heavy metal content. The suspensions were then filtered through a 0.45 μm membrane and analyzed using atomic absorption spectroscopy (Unicam 939). The pH value of the aqueous soil extracts (1:5, w/v) were measured with a glass electrode (ISO 10390:2005) followed by conductivity measurements (ISO 11265:1994).

### Microbial enumeration

The plate method was used to obtain the total oligotrophic, Zn- and Cd-tolerant culturable bacteria count. The total oligotrophic bacterial fraction was enumerated on 10% tryptic soy agar (TSA) with nystatin (50 μg/mL), while the Zn- and Cd-tolerant bacteria were counted on 10 % TSA supplemented with 1.5 mM of ZnCl_2_ and 0.5 mM of CdCl_2_, respectively. A potato-dextrose agar (PDA) medium with Rose Bengal (30 μg/mL) and streptomycin (30 μg/mL) was used for screen for fungal strains. Each were incubated for 7 days at 25°C.

### Analysis of microbial community structure by PLFA

The community structure of the non-culturable fraction of soil microorganisms was obtained by employing the phospholipid fatty acid (PLFA) analysis based on the Frostegard et al. (1993) protocol; with minor modifications. Lipids of microbial origin were extracted, fractioned, methylated and then analyzed in a GC system (Agilent 7820). Obtained FAMEs were identified and quantified using the MIDI-MIS software (Sherlock TSBA6 library; MIDI Inc., Newark, DE, USA). Calculation of total biomass, microbial biomass, biomass of Gram-positive and Gram-negative bacteria, actinomycete and fungi, all expressed as nmol PLFA g^−1^ soil d. w., was carried out based on specific lipid markers. Nonadecanoic acid (19:0) was used as an internal standard for the quantitative analysis. The procedure was described in detail in Cycon et al. (2016).

### Community-level physiological profiles (CLPPs)

The physiological diversity of microbial communities was determined using BIOLOG EcoPlates™ (Biolog Inc., CA, USA). Ten grams of dry soil was shaken with 90 mL of a sterile 0.85% saline solution for 2 h. The 96-well BIOLOG microplates were inoculated with aliquots of a 120 μL (10^−1^) dilution and incubated at 24°C for 7 days. The readings were performed every 12 h using a Bio-Tek ELx808 microplate reader at 590 nm. Absorbance measurements were corrected against the water control well and the first reading (T_0_) in order to eliminate any disturbances resulting from possible background absorbance. CLPPs were calculated by means of the corrected optical density (ODi) and area under curve values (AUC) at the 84 h reading point. This enabled conditions to be optimized and allowed comparison between plates. The pattern of the AUC corresponded to the metabolic potential of microbial communities toward the 31 substrates present on the BIOLOG EcoPlates™. Standardized AUC data was used in the Principal Component Analysis (PCA). The sum of the AUC scores (∑AUC) for individual substrates was employed as a measurement of total catabolic activity. The Shannon-Wiener index (*H'*) and evenness (*Eh*) indices were calculated as *H*' = –∑pi(lnpi) and *Eh* = *H'*/*H'*max = *H'*/ln*Rs*, respectively, where pi is the ratio of the activity on each substrate (ODi) to the sum of activities on all substrates (ΣODi) and *Rs* is the number of oxidized substrates. All analyses were performed in triplicate.

### Soil metabolomics (HPLC-MS)

Soil samples (200 mg) were placed in microcentrifuge tubes with a metal bead (5 mm diameter) and homogenized in an oscillation grinder for 10 min at 120 rpm. A 1 mL mixture of chloroform, methanol and ddH_2_0 (1:2.5:1 v/v) was added. The samples were then thoroughly mixed and centrifuged for 3 min at 5,000 × g. A supernatant was transferred to new tubes, where 0.5 mL of ddH_2_O was added and samples were centrifuged again. Two phases—polar (top) and non-polar (bottom) were separated, dried using SpeedVac® concentrator (Thermo Scientific) and stored in a freezer at −80°C. Prior to analysis, the samples were dissolved in 300 mL of 75 % methanol. From these, 20 μL of sample was diluted with 180 μL 75 % methanol, transferred to an LTQ-MS glass vial and sealed. Samples were kept at −20°C until run, in a randomized order, using an autosampler, with the tray temperature kept constant at 15°C. For each sample, 20 μL was injected into a flow volume of 60 μL per min in a ratio of 70 % water and 30 % methanol, using a Surveyor liquid chromatography system (Thermo Scientific, MA, USA). Data acquisition for each individual sample was conducted, in alternating positive and negative ionization mode, over four scan ranges (15–110 *m/z*, 100–220 *m/z*, 210–510 *m/z*, 500–1,200 *m/z*) on an LTQ linear ion trap (Thermo Electron Corporation, CA, USA), with an acquisition time of 5 min. Individual metabolite m/z values were normalized as a percentage of the total ion count for each sample. Normalized abundances were subsequently analyzed using Pychem software (Jarvis et al., 2006) to obtain PCA scatterplots. Ten major sources of variation were subjected to further statistical analysis.

### Analysis of microbial community structure by PCR-DGGE

Total DNA was extracted from 0.5 g soil samples using a GeneMATRIX Soil DNA Purification Kit (Eur_x_, Poland) as described in the manufacturer's instruction. The 16S rRNA gene fragment was amplified using the primers (GC-clamp)-F338 and R518 (Muyzer et al., 1993). Detailed information about this procedure was described in a previous paper (Cycon et al., 2013). The electrophoresis of the amplification products was performed in 8 % (w/v) polyacrylamide gel (37.5:1 acrylamide:bis-acrylamide) in the presence of a linear denaturing gradient that ranged from 40 to 70 % using a DCode Mutation Detection System (Bio-Rad, USA). In turn, a G BOX F3 System (Syngene, UK) was used to visualize the patterns of the bands that were obtained. Detailed information about the DGGE procedure was described in a previous paper (Cycon et al., 2016). The band patterns that were obtained from the DGGE analysis were analyzed using BioNumerics software ver. 7.5 (Applied Math, Belgium). The unweighted pair-group method and the arithmetic averages (UPGMA) were used to construct the phylogenic dendrograms. The biodiversity of a soil bacterial community was expressed as the Shannon-Wiener index (*H'*), richness (*Rs*) and evenness (*Eh*), which were calculated using the equations that were described in a previous paper (Cycon et al., 2013).

## Results

### Soil characteristics

The pH values obtained for tested samples were variable, however one consistent pattern could be distinguished (Table 1). The pH values were generally higher in W-derived samples (all above 7), regardless of the sample origin, with the highest score recorded in the rhizosphere of *S. vulgaris*. Against this, generally lower pH values were observed for N-derived samples achieving the lowest value in the *D. caespitosa* rhizosphere. Highly variable results were similarly obtained for total water content. The highest values of this parameter were recorded for *D. caespitosa* and *A. halleri* rhizospheres collected at the N sampling site. In contrast, the lowest total water content was noted for the rhizosphere of *S. vulgaris* (site P). The water content appeared to be strongly connected with organic matter content, as it exhibited the same pattern of variability. The results obtained for soil conductivity indicated that the highest values were from *D. caespitosa* and *A. arenosa* rhizospheres, followed by bulk soil obtained at the site W. The least conductive sample was *S. vulgaris* rhizosphere (site N) (Table 1).

**Table 1:** Physico-chemical parameters of sampled soils.

**Origin**	**Site**	**pH**	**Conductivity [μS]**	**Water content [%]**	**SOM [%]**
*Arabidopsis arenosa*	P	6.50 ± 0.20^d^	169 ± 3.61^de^	11.25 ± 1.86^f^	7.25 ± 2.22^ef^
	N	6.14 ± 0.18^e^	142 ± 9.54^g^	10.40 ± 1.62^f^	10.03 ± 1.96^cde^
	W	7.13 ± 0.13^b^	575 ± 15.04^a^	20.72 ± 2.14^c^	10.39 ± 1.41^cd^
*Arabidopsis halleri*	P	7.04 ± 0.17^ab^	165 ± 13.00^ef^	16.75 ± 1.17^d^	11.50 ± 0.49^c^
	N	5.16 ± 0.15^f^	215 ± 15.72^c^	26.06 ± 4.42^b^	15.81 ± 2.63^b^
	W	6.93 ± 0.10^ab^	190 ± 10.54^d^	13.10 ± 0.59^ef^	8.20 ± 2.00^de^
*Deschampsia caespitosa*	P	6.91 ± 0.28^ab^	143 ± 22.48^fg^	15.19 ± 2.05^de^	8.93 ± 1.92^cde^
	N	4.60 ± 0.16^g^	161 ± 6.11^efg^	32.06 ± 3.51^a^	20.90 ± 3.43^a^
	W	7.14 ± 0.11^b^	591 ± 10.60^a^	20.72 ± 2.61^c^	9.12 ± 1.09^cde^
*Silene vulgaris*	P	7.04 ± 0.16^ab^	144 ± 13.05^fg^	16.14 ± 1.12^de^	9.69 ± 0.77^cde^
	N	6.28 ± 0.18^de^	74 ± 4.16^i^	3.99 ± 0.87^g^	3.57 ± 0.96^g^
	W	7.78 ± 0.22^a^	99 ± 9.07^h^	2.92 ± 1.07^g^	2.02 ± 0.19^g^
Bulk soil	P	6.84 ± 0.07^c^	151 ± 12.58^efg^	23.84 ± 3.25^bc^	14.59 ± 1.23^b^
	N	5.27 ± 0.04^f^	98 ± 3.79^h^	10.23 ± 1.26^f^	4.63 ± 1.37^fg^
	W	7.01 ± 0.12^ab^	371 ± 29.09^b^	13.12 ± 0.90^ef^	4.64 ± 0.43^fg^

The data represent the means and standard deviations of three replicates. P, N, W indicate individual sampling sites. Different letters (within each group) indicate significant differences (P < 0.05, LSD test) considering sample origin and sampling site

Extremely high concentrations of total heavy metals, up to 38,594 and 6,021 mg kg^−1^ for Zn and Cd, respectively were measured (Table 2). The highest Zn concentration was detected in bulk soil obtained from site P. In six samples, the Zn level was higher than 20,000 mg kg^−1^ d. w. The lowest value was recorded for *D. caespitosa*, site N. Furthermore, three of the samples were found to have more than 5,000 mg kg^−1^ d. w. Cd, bulk soil, *A. arenosa* rhizosphere and *D. caespitosa* rhizosphere collected at the W sampling site. The lowest concentration of Cd was noted for bulk soil sampled at the site N. A similar pattern was observed for total Cu content, but recorded concentrations were much lower and fell within the 69–424 mg kg^−1^ d. w. range.

**Table 2:** Heavy metal content in sampled soils.

**Origin**	**Site**	**Zn tot [mg kg^−1^ d. w.]**	**Cd tot [mg kg^−1^ d. w.]**	**Cu tot [mg kg^−1^ d. w.]**	**Ni tot [mg kg^−1^ d. w.]**	**Zn bio [mg kg^−1^ d. w.]**	**Cd bio [mg kg^−1^ d. w.]**	**Cu bio [mg kg^−1^ d. w.]**	**Ni bio [mg kg^−1^ d. w.]**
*Arabidopsis arenosa*	P	8, 365 ± 570^e^	251 ± 21^ef^	69 ± 6^i^	ND ± −	350.24 ± 9.78^fg^	18.36 ± 2.03^ef^	0.14 ± 0.07^de^	*ND* ± −
	N	4, 052 ± 483^f^	143 ± 15^f^	85 ± 4^hi^	*ND* ± −	363.75 ± 16.04^f^	13.69 ± 0.91^fg^	0.18 ± 0.06^cde^	*ND* ± −
	W	31, 942 ± 3, 456^b^	5, 682 ± 715^a^	316 ± 30^b^	*ND* ± −	127.44 ± 12.13^i^	91.10 ± 2.73^b^	0.51 ± 0.13^a^	*ND* ± −
*Arabidopsis halleri*	P	28, 602 ± 2, 400^b^	1, 063 ± 188^d^	146 ± 2^de^	*ND* ± −	488.30 ± 11.12^d^	43.07 ± 12.2^d^	0.17 ± 0.06^de^	*ND* ± −
	N	5, 842 ± 231^ef^	175 ± 11^f^	159 ± 5^d^	*ND* ± −	578.14 ± 18.56^b^	23.59 ± 4.32^e^	0.28 ± 0.10^bcd^	*ND* ± −
	W	15, 969 ± 2, 930^d^	1, 687 ± 282^bc^	136 ± 4^e^	*ND* ± −	133.24 ± 11.30^i^	44.54 ± 3.54^d^	0.18 ± 0.11^cde^	*ND* ± −
*Deschampsia caespitosa*	P	20, 951 ± 2, 112^c^	762 ± 131^de^	93 ± 3^h^	*ND* ± −	432.85 ± 16.52^e^	39.50 ± 3.70^d^	0.05 ± 0.06^e^	*ND* ± −
	N	2, 572 ± 188^f^	83 ± 3^f^	114 ± 9^fg^	*ND* ± −	475.95 ± 53.16^d^	18.94 ± 3.30^ef^	0.35 ± 0.05^b^	*ND* ± −
	W	29, 682 ± 3, 008^b^	5, 735 ± 809^a^	424 ± 13^a^	*ND* ± −	87.08 ± 4.49^j^	48.23 ± 3.46^d^	0.59 ± 0.07^a^	*ND* ± −
*Silene vulgaris*	P	28, 422 ± 3, 403^b^	1, 140 ± 309^cd^	131 ± 16^ef^	*ND* ± −	534.65 ± 19.58^c^	62.78 ± 10.22^c^	0.19 ± 0.12^cde^	*ND* ± −
	N	3, 517 ± 413^f^	111 ± 11^f^	73 ± 5^i^	*ND* ± −	243.85 ± 21.26^h^	10.88 ± 2.53^fg^	0.16 ± 0.12^de^	*ND* ± −
	W	3, 581 ± 179^f^	223 ± 12^ef^	98 ± 3^gh^	*ND* ± −	11.87 ± 2.43^k^	6.32 ± 1.84^g^	0.22 ± 0.04^bcd^	*ND* ± −
Bulk soil	P	38, 594 ± 848^a^	1, 722 ± 319^b^	197 ± 10^c^	*ND* ± −	700.24 ± 52.35^a^	63.40 ± 7.22^c^	0.19 ± 0.05^cde^	*ND* ± −
	N	2, 538 ± 89^f^	77 ± 4^f^	72 ± 8^i^	*ND* ± −	319.40 ± 18.24^g^	12.18 ± 1.44^fg^	0.32 ± 0.14^bc^	*ND* ± −
	W	29, 705 ± 4, 253^b^	6, 021 ± 412^a^	313 ± 18^b^	*ND* ± −	158.54 ± 7.40^i^	102.10 ± 5.76^a^	0.32 ± 0.02^bc^	*ND* ± −

*The data represent the means and standard deviations of three replicates. P, N, W indicate individual sampling sites. Different letters (within each group) indicate significant differences (P < 0.05, LSD test) considering sample origin and sampling site (Two-way ANOVA); ND indicates that trait was not detected*.

The highest concentration of bioavailable Zn were recorded for bulk soil samples obtained at the site P, as well as rhizospheres of *A. halleri*, obtained at N sampling site. The lowest concentrations of bioavailable Zn were obtained for *S. vulgaris* and *D. caespitosa*, both of which were collected at W sampling site. The highest concentrations of bioavailable Cd were noted for the bulk soil and *A. arenosa* rhizosphere, collected at the W sampling site. The lowest level of bioavailable Cd was detected in *S. vulgaris*-derived rhizospheres obtained at site W. All tested samples had less than 1 mg kg^−1^ d. w. of bioavailable Cu. The highest concentration of the bioavailable Cu was recorded for *D. caespitosa* and *A. arenosa* rhizosphere, both from the W sampling site. The lowest value recorded for the bioavailable Cu was noted for *D. caespitosa* rhizospheres collected at the site P (Table 2).

### Microbial counts

**Figure 1:**
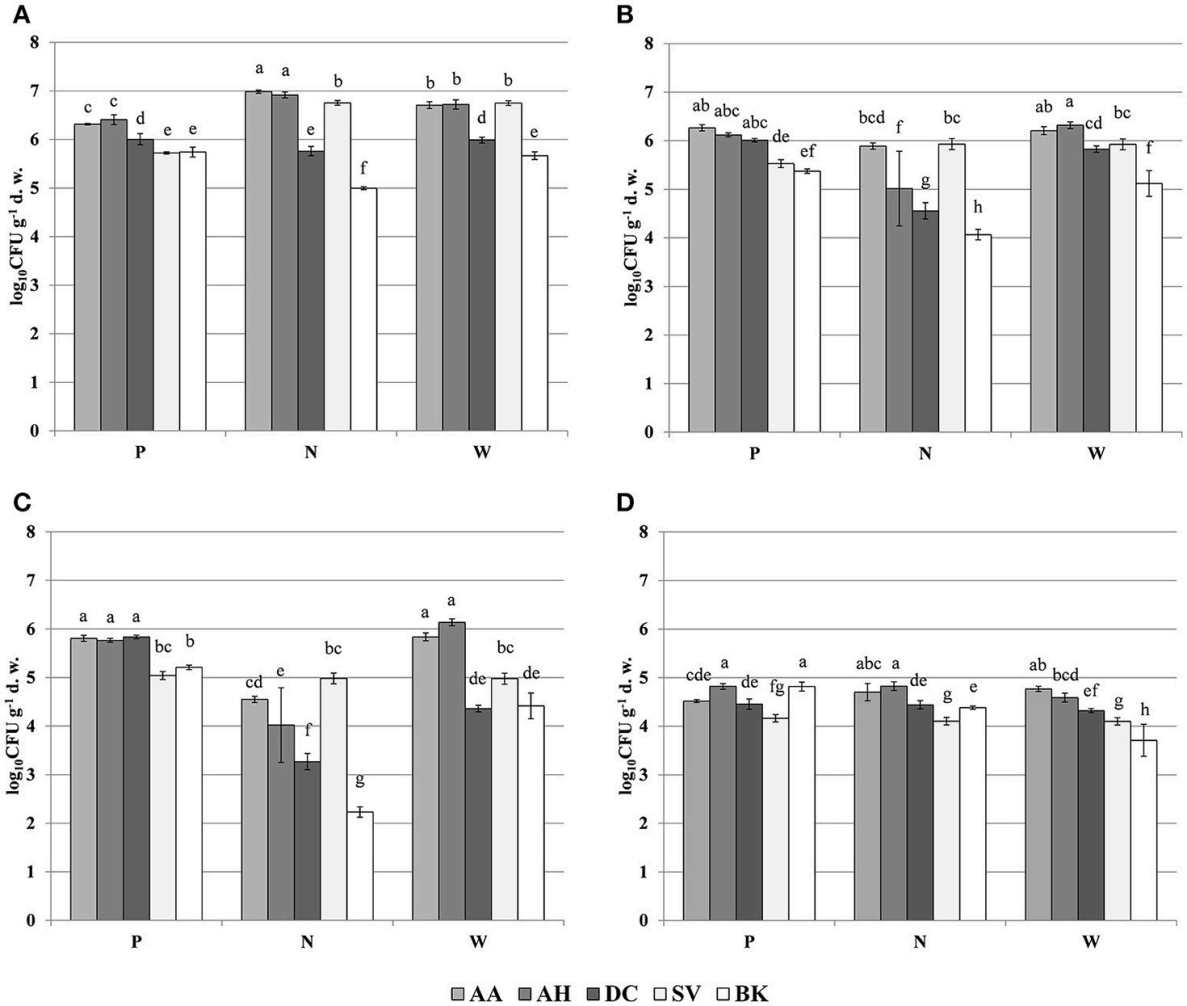
Microbial counts of: oligotrophic **(A)**, zinc-tolerant **(B)**, cadmium-tolerant **(C)** bacteria and fungi **(D)**; P, N, W, individual sampling sites; AA, *Arabidopsis arenosa*; AH, *Arabidopsis halleri*; DC, *Deschampsia caespitosa;* SV, *Silene vulgaris*; BK, bulk soil; letter designations above individual bars indicate the result of *post-hoc* LSD test of the two-way analysis of variance (*P* < 0.05).

The oligotrophic fraction of culturable bacteria generally indicated a rhizosphere effect, as for most of the plants in tested sampling sites, microbial counts were significantly lower in bulk soils. This was very pronounced for both *Arabidopsis* species, which featured increases of up to two orders of magnitudes in CFU g^−1^ d. w., when compared to bulk soil at site N. There were also differences in bacteria counts between plant species. In all of the three sampling sites, *D. caespitosa* showed significantly lower microbial counts (up to one order of magnitude lower), when compared with *Arabidopsis* species (Figure 1A). Metal-tolerant bacteria were one or even two orders of magnitude lower in number, when compared to oligotrophic fraction (Figures 1B,C). This was clearly visible especially in the Cd-tolerant fraction (Figure 1C). For oligotrophic bacteria, two-way analysis of variance indicated a strong effect of sample origin (above 69% of total variance) (Table 3). This pattern was also observed in the Zn-tolerant fraction, although an impact of sampling site and an interaction between both factors was observed (Table 3). For the Cd-tolerant fraction, sampling site appeared to have a dominant effect (Table 3). Such patterns were not observed in the fungal fraction, which were just below 10^5^ CFU g^−1^ d. w. for *A. halleri* rhizospheres obtained from sites P and N, with the lowest value observed for bulk soil at site W. However, for all three sampling sites, the *S. vulgaris* rhizospheres showed significantly lower fungal counts in comparison with other plant species (Figure 1D). Heavy metal contamination, when considered as a unique factor, had only a minor impact on microbial counts (Figure 7).

**Table 3:** Analysis of variance (two-way ANOVA) for the measured biological parameters as affected by soil origin (O), sampling site (S) and their interactions (O × S).

**Parameter**	**Source of variation**	***df***	**Sum of squares**	**Mean square**	**Variance explained (%)**	***F***	***P***
**MICROBIAL COUNTS**							
Oligotrophic bacteria (10 % TSBA + nystatin)	Origin (O)	4	9.95	2.49	69.05	479.98	<0.001^***^
	Sampling site (S)	2	0.87	0.43	6.02	83.73	<0.001^***^
	O × S	8	3.44	0.43	23.85	82.91	<0.001^***^
Zinc-tolerant bacteria (10 % TSBA + 1.5 mM Zn^2+^ + nystatin)	Origin (O)	4	8.41	2.10	41.84	41.39	<0.001^***^
	Sampling site (S)	2	6.05	3.02	30.10	59.55	<0.001^***^
	O × S	8	4.11	0.51	20.48	10.13	<0.001^***^
Cadmium-tolerant bacteria (10 % TSBA + 0.5 mM Cd^2+^ + nystatin)	Origin (O)	4	13.21	3.30	25.88	37.21	<0.001^***^
	Sampling site (S)	2	24.47	12.24	47.96	137.91	<0.001^***^
	O × S	8	10.68	1.34	20.94	15.05	<0.001^***^
Fungi (PDA + Rose Bengal + streptomycin)	Origin (O)	4	2.37	0.59	48.26	41.89	<0.001^***^
	Sampling site (S)	2	0.54	0.27	10.95	19.01	<0.001^***^
	O × S	8	1.58	0.20	32.16	13.96	<0.001^***^
**PLFA**							
Total PLFA biomass	Origin (O)	4	23,178.72	5,794.68	36.05	17.17	<0.001^***^
	Sampling site (S)	2	17,749.87	8,874.93	27.61	26.29	<0.001^***^
	O × S	8	13,233.07	1,654.13	20.58	4.90	<0.001^***^
Bacterial PLFA biomass (BB)	Origin (O)	4	3,709.42	927.36	41.83	18.09	<0.001^***^
	Sampling site (S)	2	1,520.76	760.38	17.15	14.83	<0.001^***^
	O × S	8	2,099.19	262.40	23.67	5.12	<0.001^***^
Gram-positive bacteria PLFA biomass (GP)	Origin (O)	4	500.95	125.24	26.51	7.69	<0.001^***^
	Sampling site (S)	2	550.03	275.02	29.11	16.89	<0.001^***^
	O × S	8	350.20	43.77	18.53	2.69	0.023^*^
Gram-negative bacteria PLFA biomass (GN)	Origin (O)	4	1,362.35	340.59	51.74	34.49	<0.001^***^
	Sampling site (S)	2	200.87	100.43	7.63	10.17	<0.001^***^
	O × S	8	773.77	96.72	29.38	9.79	<0.001^***^
GP:GN ratio	Origin (O)	4	0.06	0.02	3.52	1.12	0.364
	Sampling site (S)	2	0.94	0.47	54.43	34.75	<0.001^***^
	O × S	8	0.32	0.04	18.55	2.96	0.014^*^
Actinomycetes PLFA biomass	Origin (O)	4	26.73	6.68	26.82	19.26	<0.001^***^
	Sampling site (S)	2	50.31	25.16	50.50	72.52	<0.001^***^
	O × S	8	12.19	1.52	12.23	4.39	0.001^**^
Fungal PLFA biomass (FB)	Origin (O)	4	131.08	32.77	59.62	95.20	<0.001^***^
	Sampling site (S)	2	47.09	23.54	21.42	68.40	<0.001^***^
	O × S	8	31.37	3.92	14.27	11.39	<0.001^***^
BB:FB ratio	Origin (O)	4	1,129.17	282.29	44.86	107.88	<0.001^***^
	Sampling site (S)	2	542.08	271.04	21.54	103.58	<0.001^***^
	O × S	8	767.19	95.90	30.48	36.65	<0.001^***^
**BIOLOG-CLPP**							
∑AUC	Origin (O)	4	1,379,281.030	344,820.258	34.83	135.607	<0.001^***^
	Sampling site (S)	2	1,394,786.201	697,393.100	35.22	274.263	<0.001^***^
	O × S	8	1,109,553.371	138,694.171	28.02	54.544	<0.001^***^
*H'*	Origin (O)	4	0.581	0.145	21.51	5.810	0.001^**^
	Sampling site (S)	2	0.379	0.190	14.05	7.588	0.002^**^
	O × S	8	0.989	0.124	36.67	4.951	0.001^**^
*Rs*	Origin (O)	4	84.089	21.022	15.94	3.403	0.021^*^
	Sampling site (S)	2	59.511	29.756	11.28	4.817	0.015^*^
	O × S	8	198.711	24.839	37.66	4.021	0.002^**^
*Eh*	Origin (O)	4	0.022	0.005	21.72	4.76	0.004^**^
	Sampling site (S)	2	0.014	0.007	13.99	6.13	0.006^**^
	O × S	8	0.030	0.004	30.06	3.29	0.008^**^
**DGGE**							
*H'*	Origin (O)	4	0.8251	0.2063	76.10	302.0	<0.001^***^
	Sampling site (S)	2	0.1158	0.0579	10.68	85.0	<0.001^***^
	O × S	8	0.1228	0.0154	11.33	22.0	<0.001^***^
*Rs*	Origin (O)	4	2,940.8	735.2	60.83	624.2	<0.001^***^
	Sampling site (S)	2	872.1	436.1	18.04	370.2	<0.001^***^
	O × S	8	986.5	123.3	20.40	104.7	<0.001^***^
*Eh*	Origin (O)	4	0.0047	0.0012	43.63	63.0	<0.001^***^
	Sampling site (S)	2	0.0015	0.0007	13.76	40.0	<0.001^***^
	O × S	8	0.0040	0.0005	37.45	27.0	<0.001^***^

H', Shannon-Wiener biodiversity index; Rs, richness; Eh, evenness. Asterisks represent significance level according to ANOVA (

* *P < 0.05*,

** P < 0.01 and

*** *P < 0.001)*.

### PLFA analysis

PLFA differed significantly between observed parameters (Table 4). The highest values for total PLFA biomass, reaching >140 nmol PLFA g^−1^ d. w., were observed for samples obtained from the rhizosphere of *A. halleri* species at sites P and N. The lowest values were observed in the rhizospheres of *S. vulgaris* collected at the site W, as well as bulk soil samples obtained at the site N and W. A similar trend was observed for bacterial biomass where the maximal biomass was noted for the *A. halleri* rhizosphere from site N. The lowest mean value of bacterial biomass was recorded for *S. vulgaris* samples obtained from the W sampling site. This pattern extended to Gram-positive and Gram-negative bacterial biomass although the values obtained for Gram-negative bacteria were generally higher. Thus, the Gram-positive to Gram-negative bacterial ratio was lower than 1 in all of the samples, except *D. caespitosa* rhizospheres from N site where ratios were at near parity. The lowest values, linked for a major dominance of Gram-negative bacteria, were noted for the *A. arenosa, D. caespitosa, S. vulgaris* and bulk soil samples from site W. The highest actinomycete biomass was recorded for rhizospheres obtained from *A. arenosa* and *A. halleri* collected from the P sampling site, while the lowest value was noted for bulk soils obtained from site W. Considering fungal biomass, the highest measurement was recorded for the rhizosphere of *A. arenosa* from site P. The lowest values were measured in the rhizosphere of *D. caespitosa* from N sampling site and bulk soil samples obtained at sites W and N. The highest bacterial to fungal biomass ratio was obtained for the samples collected from the *D. caespitosa* rhizosphere at the site P, whereas the lowest was recorded for the *A. arenosa*-derived samples obtained at the P sampling site. Generally, low values were also recorded for samples obtained from *S. vulgaris* rhizospheres.

**Table 4:** PLFA biomass (nmol PLFA g^−1^ d. w.) parameters as affected by soil origin (O), sampling site (S) and their interactions.

**Origin**	**Site**	**Total PLFA**	**Bacterial PLFA**	**Bacteria**		**GP:GN**	**Actinomyctetes**	**Fungi**	**Bacteria:fungi**
				**Gram-positive (GP)**	**Gram-negative (GN)**				
AA	P	138.63 ± 30.00^a^	43.54 ± 10.72^ab^	19.62 ± 6.45^ab^	21.92 ± 3.84^bcd^	0.88 ± 0.16^ab^	5.63 ± 0.63^ab^	9.49 ± 1.17^a^	4.54 ± 0.60^j^
	N	104.35 ± 11.84^ab^	36.26 ± 3.70^b−e^	16.05 ± 3.02^b−e^	18.29 ± 0.84^cde^	0.87 ± 0.15^ab^	3.25 ± 0.63^cd^	4.13 ± 0.31^c^	8.81 ± 1.04^fg^
	W	103.93 ± 31.37^ab^	34.86 ± 11.11^b−e^	10.61 ± 4.71^d−g^	22.74 ± 6.06^bc^	0.45 ± 0.11^f^	2.70 ± 0.66^cde^	5.62 ± 1.29^b^	6.11 ± 0.65^hij^
AH	P	143.64 ± 11.54^a^	46.25 ± 4.27^b^	17.80 ± 2.59^bc^	26.52 ± 1.52^b^	0.67 ± 0.06^cde^	5.84 ± 0.83^a^	6.07 ± 0.36^b^	7.61 ± 0.37^f−i^
	N	145.08 ± 29.83^a^	62.74 ± 14.81^a^	24.57 ± 8.72^a^	36.08 ± 6.49^a^	0.67 ± 0.16^cde^	3.48 ± 0.25^c^	3.52 ± 0.76^cd^	17.87 ± 2.54^b^
	W	90.26 ± 19.25^c^	32.20 ± 7.32^cde^	12.06 ± 3.42^c−g^	18.73 ± 3.61^cde^	0.64 ± 0.07^def^	2.48 ± 0.37^de^	4.37 ± 0.57^c^	7.30 ± 0.79^ghi^
DC	P	74.58 ± 11.75^cd^	25.19 ± 4.43^ef^	9.53 ± 2.67^e−h^	14.65 ± 2.23^efg^	0.65 ± 0.16^cde^	2.60 ± 0.41^cde^	2.54 ± 0.48^de^	10.04 ± 1.60^ef^
	N	88.90 ± 25.43^c^	38.84 ± 10.89^bcd^	18.94 ± 6.91^ab^	18.43 ± 3.73^cde^	1.01 ± 0.21^a^	2.05 ± 0.39^ef^	1.12 ± 0.34^f^	34.80 ± 2.35^a^
	W	77.83 ± 15.60^cd^	28.19 ± 5.94^def^	9.03 ± 2.93^f−h^	18.00 ± 2.86^cde^	0.49 ± 0.10^ef^	2.00 ± 0.24^ef^	1.92 ± 0.16^ef^	14.68 ± 3.07^c^
SV	P	103.25 ± 2.66^ab^	31.81 ± 1.65^cde^	13.19 ± 0.54^b−f^	16.83 ± 1.28^def^	0.79 ± 0.02^bcd^	3.14 ± 0.13^cd^	4.02 ± 0.45^c^	8.01 ± 1.41^fgh^
	N	76.13 ± 9.45^cd^	24.45 ± 2.99^ef^	10.60 ± 1.84^d−g^	12.44 ± 1.02^fgh^	0.85 ± 0.09^abc^	2.40 ± 0.40^de^	4.25 ± 0.20^c^	5.76 ± 0.67^hij^
	W	29.35 ± 4.40^e^	9.04 ± 1.82^g^	2.89 ± 0.70^h^	5.75 ± 1.08^i^	0.50 ± 0.07^ef^	1.31 ± 0.98^fg^	1.85 ± 0.49^ef^	4.97 ± 0.60^ij^
BK	P	133.03 ± 18.84^ab^	40.16 ± 4.74^ab^	16.71 ± 2.16^bcd^	21.41 ± 2.31^bcd^	0.78 ± 0.03^bcd^	4.72 ± 0.78^b^	3.42 ± 0.18^cd^	11.73 ± 0.80^de^
	N	49.94 ± 4.81^de^	18.26 ± 1.43^fg^	8.62 ± 1.01^f−h^	8.72 ± 0.42^hi^	0.99 ± 0.10^a^	2.07 ± 0.98^ef^	1.33 ± 0.14^f^	13.84 ± 1.33^cd^
	W	48.65 ± 11.14^de^	18.05 ± 3.59^fg^	6.18 ± 1.87^g−h^	10.94 ± 1.46^ghi^	0.56 ± 0.10^ef^	0.76 ± 0.06^h^	1.31 ± 0.32^f^	14.08 ± 2.73^cd^

*The data represent the means and standard deviations of three replicates. Different letters (within each group) indicate significant differences (P < 0.05, LSD test) considering sample origin and sampling site (Two-way ANOVA). AA, Arabidopsis arenosa; AH, Arabidopsis halleri; DC, Deschampsia caespitosa; SV, Silene vulgaris; BK, bulk soil*.

Most of the tested parameters, especially the quantitative parameters, were found to be associated more with sample origin (i.e., rhizosphere vs. bulk soil) over sampling site although interactions between sample origin and site were occasionally significant. In case of total PLFA biomass, sampling origin explained 36.05 % of variance and in the case of fungal biomass up to 59.62 % of variance. For three of the tested PLFA parameters, Gram-positive bacterial biomass, Gram-positive to Gram-negative bacterial biomass ratio and actinomycete biomass, the sampling site turned out to be the major source of variation (Table 3).

**Figure 2:**
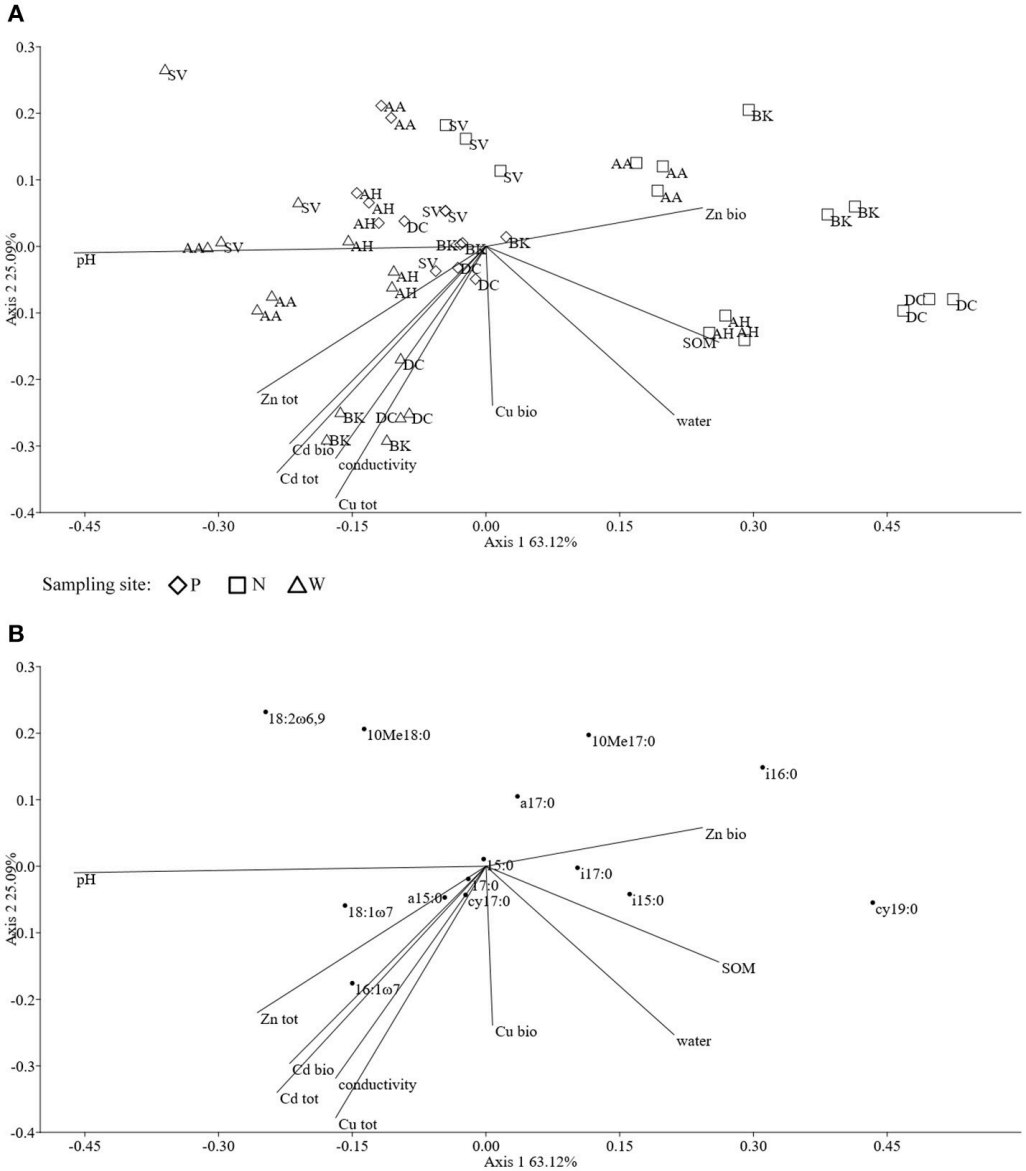
The ordination plot of CCA for standardized PLFA biomarker profiles; biplot of samples (symbols) and environmental variables (lines) **(A)**; biplot of fatty acids (dots) and environmental variables (lines) **(B)**.

The ordination plot of CCA for standardized PLFA profiles showed a distinct clustering of the samples along axis 1 (Figure 2A). Three major clusters could be distinguished and were related to individual sampling sites (P, N, and W). There was a strong correlation between pH and sample scattering along axis 1. Additionally, a significant effect of total Cd, Zn, Cu, conductivity as well bioavailable Cd was seen in the PLFA profiles (Figure 7). This corresponded with the bulk soil and rhizospheres of *D. caespitosa*, obtained at site W (Figure 2A). Additionally, there was a correlation between these parameters and 16:1ω7 as well as 18:1ω7 fatty acids (Figure 2B). Interestingly, the impact of bioavailable Zn is differed from that of bioavailable Cd, Cu or total Cd, Zn, and Cu concentrations. Therefore Zn could act as a stimulant for certain microbial groups, as opposed to Cd.

### CLPP analysis

**Figure 3:**
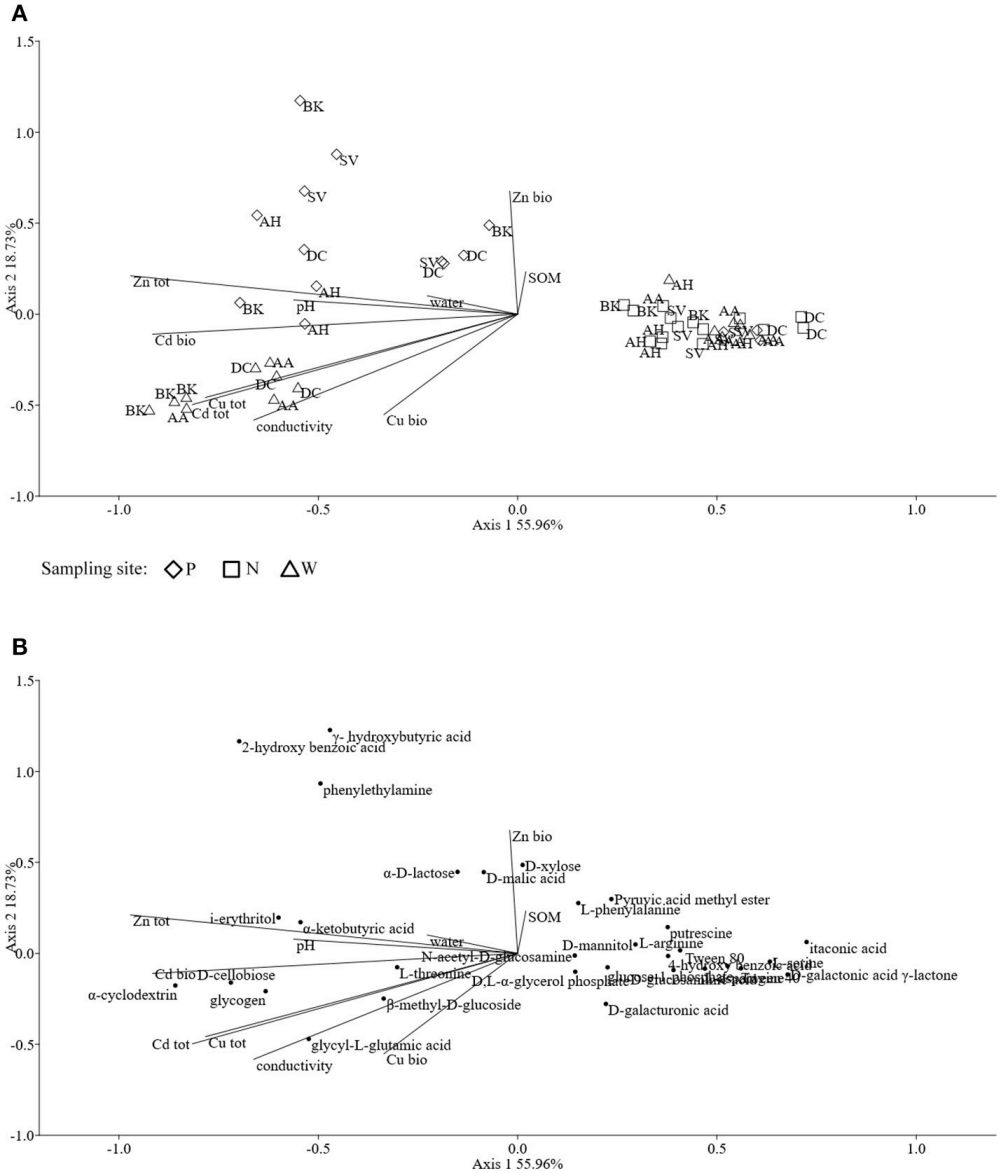
The ordination plot of CCA for standardized AUC profiles of 31 substrates; biplot of samples (symbols) and environmental variables (lines) **(A)**; biplot of substrates (dots) and environmental variables (lines) **(B)**.

Assessments based on the BIOLOG method showed that the catabolic activity of the microbial communities varied significantly for individual microbial communities (Table 5). The highest values of total catabolic activity (∑AUC) were recorded for the rhizosphere samples of *A. halleri* and *S. vulgaris*, from site N. High values were also obtained for rhizospheres from the same species at the site W, as well as the rhizosphere of *A. arenosa* collected at the site P. The lowest catabolic activity was observed for the rhizosphere of *S. vulgaris* and bulk soil samples obtained at the site P and W. Generally a higher ∑AUC score was noted for rhizosphere samples in comparison with the bulk soil equivalents obtained from the same sampling sites, indicating a distinctive rhizosphere effect. Similar patterns were obtained for the Shannon-Wiener biodiversity (*H'*) as well as evenness (*Eh*) indices, reaching peak values for *A. arenosa*-derived samples at site N and the lowest for bulk soil from site W. The most active microbial communities in terms of the total number of oxidized substrates (*Rs* index), were derived from the rhizospheres of *A. arenosa* and *A. halleri*, site N and W. The lowest score was again noted for the bulk soil, site W (Table 5). The two-way ANOVA showed that all of the factors were almost equally explanatory to the total variance observed for the ∑AUC with regard to sampling site, sample origin and interactions between both factors. The other parameters were mostly affected by the interaction of sample origin and sampling site. Even though the effect of sample origin and sampling site was less pronounced, the impact of those factors on CLPP indices was statistically significant (Table 3). The ordination plot of standardized AUC profiles of individual substrates showed a distinct scattering of samples mainly along axis 1, explaining 55.96 % of total variance (Figure 3A). A distinct, tight cluster of *D caespitosa, A. arenosa* rhizospheres as well as bulk soils derived from site W can be observed. Overall, the separation could be related to heavy-metal concentration and was most influenced by total Cd and Zn as well as bioavailable Zn. Total Cu content, conductivity or pH proved to be minor contributors. The CCA biplot of individual substrates and environmental variables showed, that the oxidation of α-cyclodextrin, D-cellobiose, glycogen and glycyl-L-glutamic acid by microbial communities was significantly affected by the presence of heavy metals (Figure 3B). This indicates a significant effect of heavy metal contamination on microbial CLPPs. The significance of this effect was also confirmed by a strong negative correlation between BIOLOG indices and total contents of Zn, Cd, and Cu, as well as bioavailable Cd (Figure 7).

**Table 5:** BIOLOG parameters as affected by soil origin (O), sampling site (S), and their interactions.

**Origin**	**Site**	**ΣAUC**	***H'***	***Rs***	***Eh***
*Arabidopsis arenosa*	P	454.35 ± 13.21^c^	2.98 ± 0.06^ab^	29.00 ± 1.00^a^	0.88 ± 0.02^abc^
	N	660.19 ± 43.57^b^	3.06 ± 0.03^a^	27.67 ± 1.53^abc^	0.92 ± 0.01^a^
	W	174.09 ± 54.67^de^	2.57 ± 0.28^c^	24.33 ± 5.03^cd^	0.81 ± 0.05^ef^
*Arabidopsis halleri*	P	110.40 ± 59.36^efg^	2.56 ± 0.31^c^	21.00 ± 6.00^de^	0.85 ± 0.02^bcde^
	N	885.88 ± 65.27^a^	2.95 ± 0.03^ab^	28.67 ± 0.58^ab^	0.88 ± 0.01^abc^
	W	677.57 ± 88.56^b^	2.98 ± 0.05^ab^	29.00 ± 1.00^a^	0.88 ± 0.01^abc^
*Deschampsia caespitosa*	P	86.43 ± 34.75^fg^	2.97 ± 0.36^ab^	27.33 ± 3.51^abc^	0.90 ± 0.09^ab^
	N	375.26 ± 57.17^c^	2.76 ± 0.04^bc^	27.00 ± 0.00^abc^	0.84 ± 0.01^cde^
	W	248.11 ± 18.75^d^	2.66 ± 0.06^c^	25.33 ± 0.58^abc^	0.82 ± 0.01^def^
*Silene vulgaris*	P	32.47 ± 1.47^g^	2.79 ± 0.17^bc^	24.67 ± 1.53^bcd^	0.87 ± 0.04^abcd^
	N	817.98 ± 75.70^a^	3.00 ± 0.05^ab^	28.00 ± 1.00^abc^	0.90 ± 0.01^ab^
	W	659.82 ± 80.76^b^	2.94 ± 0.04^ab^	27.00 ± 1.00^abc^	0.89 ± 0.00^ab^
Bulk soil	P	53.88 ± 14.96^g^	2.65 ± 0.06^c^	24.00 ± 2.00^cd^	0.83 ± 0.03^cde^
	N	154.43 ± 12.01^ef^	2.80 ± 0.08^abc^	26.33 ± 1.15^abc^	0.86 ± 0.01^bcde^
	W	58.43 ± 1.69^g^	2.29 ± 0.12^d^	19.33 ± 2.08^e^	0.78 ± 0.05^f^

*The data represent the means and standard deviations of three replicates. Different letters (within each group) indicate significant differences (P < 0.05, LSD test) considering sample origin and sampling site (Two-way ANOVA). ∑AUC, summed area under the curve for all the substrates; H', Shannon-Wiener index; Rs, richness; Eh, evenness*.

### HPLC-MS analysis

**Figure 4:**
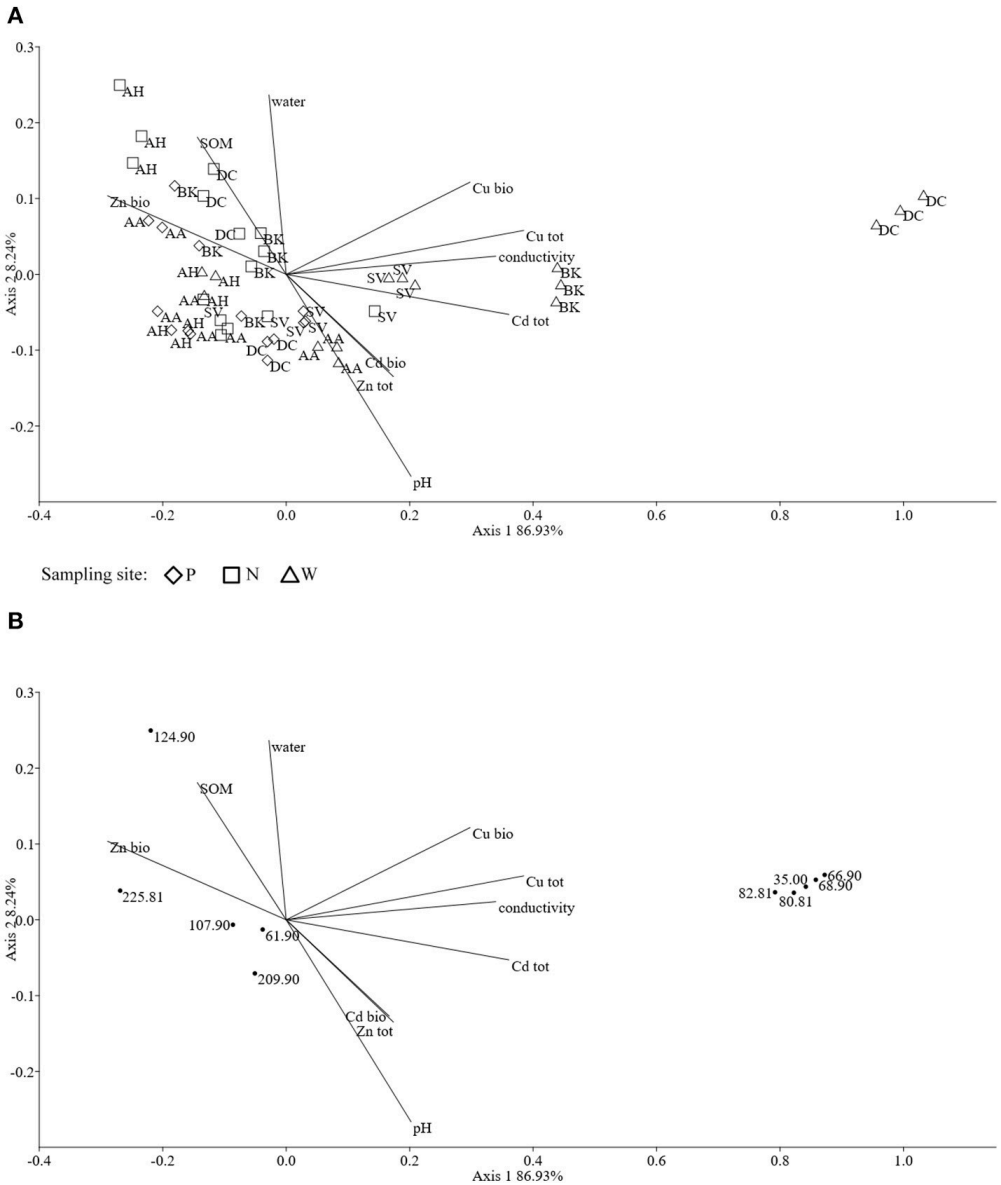
The ordination plot of CCA for standardized HPLC-MS profiles; biplot of samples (symbols) and environmental variables (lines) **(A)**; biplot of metabolite *m/z* (dots) and environmental variables (lines) **(B)**.

The obtained MS data was subjected to PCA (data not shown). Ten identified major sources of variation (out of 2,106 variables) were used for CCA in order to detect any links between soil physico-chemical parameters and MS data. The CCA biplot of samples and soil environmental data showed a distinct scattering that was found to be impacted mostly by soil pH, however this was mostly along axis 2, explaining only just above 8 % of total variation. However, an impact of the presence of heavy metals, in both total and bioavailable fractions, was seen for *D. caespitosa* rhizosphere and bulk soil (site W). They were correlated with total Cd as well as both total and bioavailable Cu, as well as soil conductivity and separated along axis 1 which explained 87 % of total variation (Figure 4A). The metabolites associated with these environmental variables were of low molecular weight (35.00, 66.90, 68.90, 80.81, and 82.81 *m/z*). Database comparisons based on accurate mass, led to their tentative identification (Table 6; Figure 4B). The *A. arenosa* rhizosphere obtained from the W sampling site correlated with soil pH and bioavailable Cd. Interestingly, the *A. halleri* rhizosphere samples separated in the opposite direction, to the left of axis 1, regardless of the sampling site, and correlated with 124.90 and 225.081 *m/z* metabolites that could potentially correspond to arsenous acid, formic acid and indole, 3-(2-aminoethyl)-7-methoxy-, hydrochloride or 2,2,4-trimethyl-1,3-pentanediol, respectively (Table 6; Figures 4A,B).

**Table 6:** Metabolite candidates for obtained *m/z* peaks.

**Metabolite name**	**Molecular formula**	**Mass (*m/z*)**		**Adduct delta**
		**Detected**	**Accurate**	
No data	–	35.00	–	–
No data	–	61.90	–	–
Hydrazine^*^	H_4_N_2_	66.90	67.007 [M+Cl]1-	0.107
Pyrazole^*^	C_3_H_4_N_2_		67.030 [M-H]1-	0.130
Methanol^*^	C_4_H0		66.996 [M+Cl]1-	0.096
Imidazole^*^	C_3_H_4_N_2_		67.030 [M-H]1-	0.130
3-Butyn-2-ol^*^	C_4_H_6_O	68.90	69.035 [M-H]1-	0.135
Propynoic acid^*^	C_3_H_2_O_2_		68.998 [M-H]1-	0.098
Beta-aminopropionitrile^*^	C_3_H_6_N_2_		69.046 [M-H]1-	0.146
2-Butyn-1-ol^*^	C_4_H_6_O		69.035 [M-H]1-	0.135
3-Butyn-1-ol^*^	C_4_H_6_O		69.035 [M-H]1-	0.135
Formic acid^*^	CH_2_O_2_	80.81	80.975 [M+Cl]1-	0.165
Sulfurous acid^*^	H_2_O_3_S		80.965 [M+Cl]1-	0.155
Methyl hydroperoxide^*^	CH_4_O_2_	82.81	82.991 [M+Cl]1-	0.181
Methanethiol^*^	CH_4_S		82.973 [M+Cl]1-	0.163
Chloric acid^**^	HClO_3_		82.954 [M+Cl]1-	0.144
No data	–	107.90	–	–
Arsenous acid^*^	H_3_AsO_3_	124.90	124.923 [M-H]1-	0.023
Formic acid^*^	CH_2_O_2_		124.924 [M+Br]1-	0.024
No data	–	209.90	–	–
Indole, 3-(2-aminoethyl)-7-	C_11_H_15_ClN_2_O	225.81	225.080 [M-H]1-	−0.001
methoxy, hydrochloride^*^				
2,2,4-trimethyl-1,3-pentanediol^*^	C_8_H_18_O_2_		225.050 [M+Br]1-	−0.031

Data origin:

* *https://dimedb.ibers.aber.ac.uk/*;

** *Individual accurate mass assessment*.

### PCR-DGGE analysis

**Figure 5:**
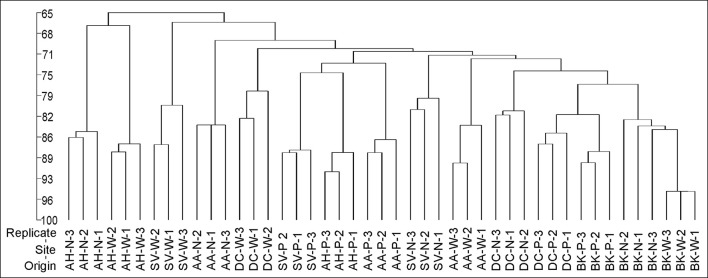
The phylogenic dendrogram for PCR-amplified fragments of the 16S rRNA gene generated on the basis of DGGE profiles obtained for the rhizosphere or bulk soil collected from different sites; P, N, W, individual sampling sites; AA, *Arabidopsis arenosa*; AH, *Arabidopsis halleri*; DC, *Deschampsia caespitose*; SV, *Silene vulgaris*; BK, bulk soil.

**Figure 6:**
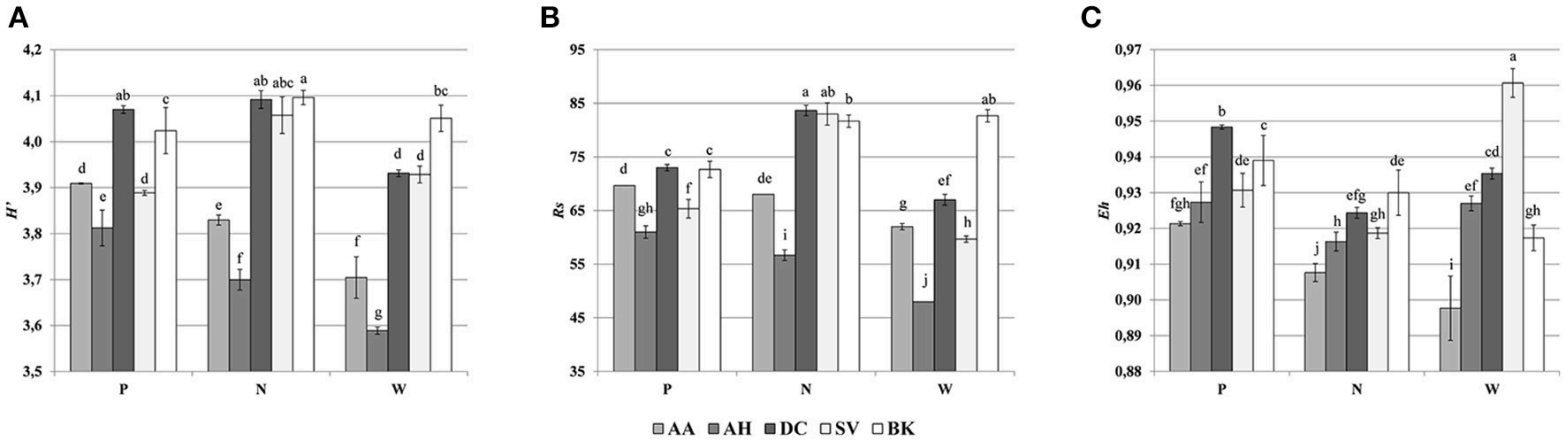
Shannon-Wiener index **(A)**, richness **(B)**, evenness **(C)**, determined on the basis of DGGE profiles obtained for the rhizosphere or bulk soil of plants collected from different sites; P, N, W, individual sampling sites; AA, *Arabidopsis arenosa*; AH, *Arabidopsis halleri*; DC, *Deschampsia caespitosa*; SV, *Silene vulgaris*; BK, bulk soil; letter designations above individual bars indicate the result of *post-hoc* LSD test of the two-way analysis of variance (*P* < 0.05).

Cluster analysis of the DGGE profiles revealed a mixed pattern for the structure of dominant bacterial communities (Figure 5). The major source of variation (sampling site or sample origin) could not be identified solely based on the clustering pattern itself, therefore, biodiversity indices were also analyzed. The genetic diversity (*H'*), richness (*Rs*) and evenness (*Eh*) indices showed significant differences between tested samples (Figure 6). A distinct, origin-dependent pattern can be observed for both, *H'* and *Rs* indices. Among the obtained rhizospheres, the highest values of *H'* and *Rs* indices were noted for samples derived from *D. caespitosa*, while the lowest were linked to *A. halleri*, regardless of sampling site. Surprisingly, the bulk soil did not show a typical rhizosphere-effect pattern. The *H'* and *Rs* indices were relatively high, reaching values comparable with those obtained for *D. caespitosa* rhizospheres or even higher for samples obtained at site W (Figure 6). Two-way ANOVA clearly showed, that sample origin accounted for the majority of total variation observed (Table 3). The highest values for *Eh* index were recorded for *D. caespitosa* only in samples from P and N sampling sites. However, the lowest values of this index were noted in samples associated with *A. arenosa*. Two-way ANOVA showed that sample origin as well as the interaction between sample origin and sampling site explained most of the total variance observed in the *Eh* index (Table 3).

**Figure 7:**
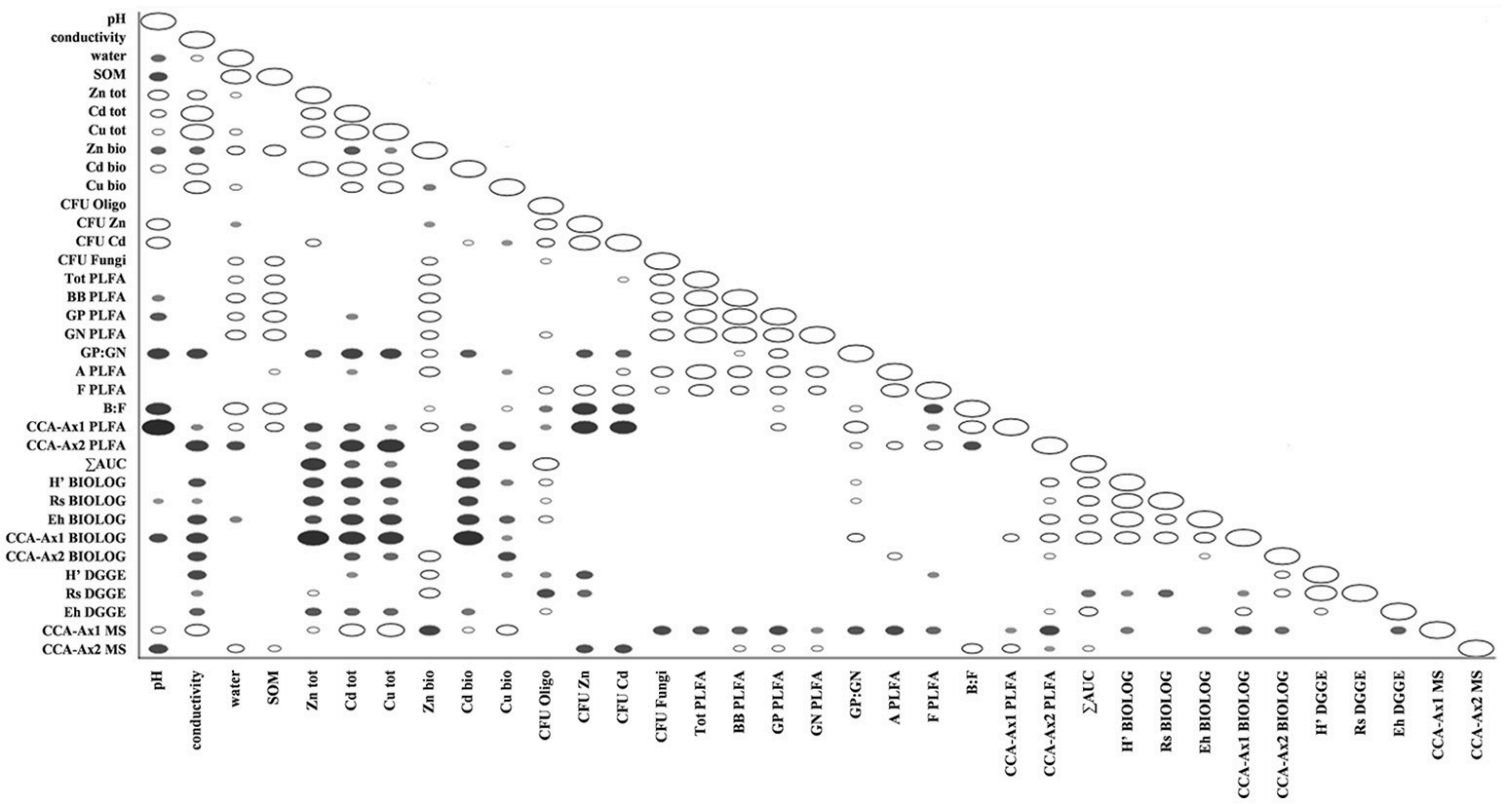
Spearman correlations between obtained physico-chemical and biological parameters in tested soils; filled markers correspond to negative correlations; hollow markers correspond to positive correlations (*P* < 0.05); marker size corresponds to correlation strength.

The Spearman correlation analysis of PCR-DGGE indices with environmental variables showed a significant negative impact of conductivity on all of the indices. This was in accord with the negative correlation found between the *Eh* index and total fractions of Zn, Cd and Cu as well as bioavailable Cd. Additionally, the bioavailable Cd negatively affected the *H'* index as well. However, *H'* and *Rs* indices were positively correlated with bioavailable Zn. This suggests a stimulative effect of bioavailable Zn on the microbial composition, whereas the effect of Cd was clearly adverse (Figure 7).

Datasets obtained via individual techniques provided unique information regarding specific microbial traits, such as microbial counts, structural, functional and genetic biodiversity, as well as metabolomic profiling. Spearman correlation analysis revealed only a slight positive correlation for ∑AUC and richness index obtained from DGGE, as well as ∑AUC and microbial counts for oligotrophic microbial fraction (Figure 7).

## Discussion

The urgent requirement to deal with the impact of environmental pollution with heavy metals demands that a more thorough understanding of how plants interact with the rhizosphere microbes in contaminated soils. In this study, we extensively characterized the rhizosphere microbial communities of *Arabidopsis arenosa, Arabidopsis halleri, Deschampsia caespitosa, Silene vulgaris* as well as bulk soils with high heavy metal content. These species could be classified to a group broadly referred to as pseudometallophytes (Baker et al., 2010; van der Ent et al., 2013). Nonetheless, multiple classification systems coexist and therefore a definite assignment for certain metal-tolerant plants is difficult. For instance, *A. halleri* is a known zinc and cadmium hyperaccumulator, yet some populations of this species are known to occur in non-metalliferous sites (Pauwels et al., 2006; Meyer et al., 2010). Furthermore, the exact functionality of such species is likely to be dependent on and therefore reflect, associated microbial communities. Therefore, in this study, we concentrated not only on the structure but crucially on some functional aspects of differing microbial populations. These could define the ability of a particular plant species to survive on heavy metal contaminated soil.

In our study, rhizosphere microbial communities reached significantly higher microbial counts for both total oligotrophic bacteria fractions as well as metal-tolerant ones. As demonstrated in many previous studies (Houlden et al., 2008; Oliveira et al., 2008), microbial counts were positively affected by the presence of a plant. This phenomenon, known as the rhizosphere effect, arises from the release of plant-derived metabolites that serve as carbon source for various microorganisms (Bais et al., 2006; Hartmann et al., 2009; Hinsinger et al., 2009). Plants might also recruit certain groups of microorganisms which are useful to them. Rudrappa et al. (2008) showed, that L-malic acid secreted by *Arabidopsis thaliana* roots recruited *Bacillus subtilis* which had beneficial effect on disease-affected plants. Below-ground microflora modifications in response to whitefly infestation of *Capsicum annuum*, contributed to the exhibition of systemic resistance and improved plant growth (Yang et al., 2011).

The results obtained for the metal-tolerant microbial counts can be influenced by the choice of growth medium, especially when defining microbial resistance to specific metal. Between many components of growth media, such as phosphates, broth components, organic acids and metals, an interaction may occur. In fact, up to 90% of the applied concentration of metal ions may be complexed by the medium and this effect is especially prominent in broth containing media, which are considered “rich” (Mergeay, 1995). To mitigate this effect, in our study a diluted 10 % concentration of tripticase soy broth agar (TSBA) was used. Broth media, such as nutrient agar (NA), Luria-Bertani (LB) and TSBA are still used in metal tolerance assays, along with substrate-amended mineral media, like 284 medium (Piotrowska-Seget et al., 2005; Freitas et al., 2008; Becerra-Castro et al., 2012). Although it cannot be considered as bias-free, the selected concentrations of 1.5 mM Zn^2+^ and 0.5 mM Cd^2+^ used in our work, resulted in lower metal-tolerant bacterial counts in comparison with oligotrophic fraction obtained on a metal-free 10% TSBA, which indicates the limiting effect of metal supplementation.

Our study demonstrated a significant impact of plant species on the genetic diversity of microbial communities, as revealed by 16S rRNA PCR-DGGE analysis. The role of plants in the determination of microbial communities was also shown by Chaparro et al. (2014), who related root exudates at four developmental stages of *Arabidopsis thaliana* with changes in microbial rhizosphere communities. They observed significant differences in the microbial community structure during plant development, especially between the seedling and vegetative, bolting and flowering stages. In our study, individual plant species showed specific patterns of DGGE biodiversity indices. This effect of plant species was significant and the differences could be distinguished even between *A. halleri* and *A. arenosa*, regardless the sampling site or heavy metal content. This accorded with a study conducted by Wang et al. (2008), who examined a potential effect of copper on soil microbial activity and community composition in samples derived from Cu-accumulator, *Elsholtzia splendens*, and a non-Cu-accumulator plant, *Trifolium repens*. Soil microbial biomass and phosphatase activity in the *E. splendens* rhizosphere were higher than those of *T. repens*. Also the PCR-DGGE fingerprint analysis showed that Cu decreased the number of bands in bare soil and soil with *T. repens*, but significantly increased the number of bands in Cu-amended soils with *E. splendens*. In our work, a significant difference in PCR-DGGE biodiversity indices and band numbers was observed even between the soils derived from a hyperaccumulator *Arabidopsis halleri* and non-hyperaccumulator *Arabidopsis arenosa*. The literature shows that the rhizobiome of hyperaccumulators is likely to contain of specific Gram-positive genera (Melo et al., 2011; Visioli et al., 2015). Similarly, Luo et al. (2017), found a significant impact of Cd/Zn hyperaccumulating and non-hyperaccumulating genotypes of *Sedum alfredii* on the plant-associated microbial community structure. The rhizosphere of hyperaccumulating plant also showed lower total OTU counts than non-hyperaccumulating one. In our work, *A. halleri* rhizospheres featured the lowest Shannon-Wiener index as well as DGGE band counts than any other rhizosphere or bulk soil.

This need not indicate that plant species alone influences microbial population. Schlaeppi et al. (2014) revealed that the composition of rhizosphere microbial communities was influenced more by wide array of environmental parameters. Zhang et al. (2016) examined the effects of heavy metals and soil physicochemical properties on microbial biomass and structure in wetlands and saw a significant impact of heavy metals. However, it seems likely that plant and metals interact to influence rhizosphere microbiology. Wood et al. (2016) observed a significant impact of both cadmium concentration and plant on the Shannon diversity index of obtained OTU profiles in the rhizosphere microbial communities of a Cd accumulator *Carpobrotus rossii*.

Microbial community structure can also be characterized on the basis of biochemical markers, such as PLFAs. This method relies on the fact, that some PLFAs are characteristic of certain microbial groups, such as Gram-positive and Gram-negative bacteria, actinomycete as well as fungi (Frostegard et al., 1996). Impact of plants on PLFA profiles was demonstrated through administration of ^13^C-labeled mixture of glucose, glycine and fumaric acid into soil in proportions resembling the exudate profile of young *Lolium perenne* plants (Paterson et al., 2007). There were significant changes in the Gram-positive and Gram-negative bacteria, fungi and actinomycete biomass for each substrate, thus proving the major impact of rhizodeposition on the microbial community structure (Paterson et al., 2007). The effect of plant exudates on the structural biodiversity of soil microbiota was also demonstrated by Kozdrój and Van Elsas (2000) in soils contaminated with heavy metals. A dominant effect of the plant on microbial community structure was observed by Pacwa-Płociniczak et al. (2018) in a study regarding the effect of *Silene vulgaris* and Cd presence on soil microbiota. Against this, in our work, no impact of plant species on microbial PLFA profiles was observed. This may result from the fact, that soil microbial communities in the environment were subjected to physical or chemical stimuli that could have masked the effect of plant exudates. In this context, water content and pH are known to affect soil microbial communities to a great extent and our work clearly showed the impact of pH on PLFA biomarkers. Shifts in pH, water and organic matter content can effectively influence the bioavailable heavy metal concentration (Rieuwerts et al., 1998; Gadd, 2004, 2010).

Several studies have indicated that heavy metals might be an important factor affecting rhizosphere microbial communities (Frostegard et al., 1996; Frostegård et al., 2011; Li et al., 2017). For instance, a significant impact of As on paddy soils was shown using the PLFA analysis of rhizospheres derived from rice plants (Li et al., 2017). In particular, biomarkers associated with Gram-negative bacteria, such as 16:0, 16:1ω7c, 16:1ω9c, 18:1ω7c, and 18:1ω9c was shown to increase with the presence of heavy metals. In our study, we also observed a strong influence of Cd as well as sampling site on the PLFA microbial profiles. Shifts between Gram-positive and Gram-negative bacterial biomarkers were noted. In particular, 16:1ω7 was identified as the major source of variation and was strongly associated with total and bioavailable Cd as well as total Zn. It is worth noting, that heavy metal-dependent variations were observed mainly in qualitative data, such as PLFA profiles, rather than quantitative, like PLFA biomass.

To examine if plant-derived metabolites might affect microbial abundance and activity, the metabolic potential of different microbial communities was measured using BIOLOG-CLPP (Martínez-Iñigo et al., 2009). Earlier studies using CLPP profiling of microbial communities derived from bulk soil as well as *Dactylis glomerata, Phalaris arundinacea, Phleum pretense*, and *Trifolium pratense* rhizospheres demonstrated a significant effect of plant species on the microbial metabolic potential (Söderberg et al., 2004). This effect surpassed even the impact of different soils used in the experiment. The Söderberg et al. (2004) study however did not include heavy-metal contamination as additional variable, which was important factor in our work. In this regard, Li et al. (2011) found no significant relationship between the functional diversity indices and heavy-metal content. Instead, a significant impact of *Erigeron annuus* and *Lysimachia clethroides* plants species on CLPP profiles was demonstrated. Similar results were obtained in a study on Ni phytoextraction in serpentine soils, where the phenotypic structure of the bacterial communities appeared to be specific to the plant cover (Rue et al., 2015). Nevertheless, other studies have suggested heavy metals have significant effects on the microbial CLPPs (Stefanowicz et al., 2008). In our study, we observed a drop in microbial metabolic activity with exposure to heavy metals. The tested soils were heavily contaminated with heavy metals, especially Zn and Cd. Cd, in particular, being a non-essential metal, is considered to be significantly more toxic than Zn or Cu (Guo et al., 2010; Clemens and Ma, 2016; Sobariu et al., 2017).

To help define the exact nature of the plant exudates that could be influencing microbial communities, metabolomic approaches were adopted. These analyses revealed that several compounds of the same molecular mass were found in *Arabidopsis* species. Phenolic compounds are recognized as common *Arabidopsis* exudates that modulate rhizosphere microbial communities (Badri et al., 2013). Tentative identification only was possible in our study but several other low-molecular compounds were noted in samples containing Cd.

Considering known responses of plant metabolism to heavy-metal toxicity, various compounds of known antioxidative properties are produced such as carotenoid-related xanthophylls, glutathione or ascorbic acid. Some secondary metabolites, such as polyphenols (flavonoids, especially anthocyanins) act against reactive oxygen species (Sytar et al., 2013). Organic acids, such as malate, citrate and oxalate might play an important role in metal binding in plants, especially in the roots (Rascio and Navari-Izzo, 2011; Sytar et al., 2013). One potential metabolite recognized in our study was methyl hydroperoxide, which could indicate oxidative stress. Cieślinski et al. (1998) in a study on the Cd accumulation in the *Triticum turgidum* var. *durum* plants showed a strong relationship between extractable soil Cd and low-molecular organic acids, such as oxalic, fumaric, succinic, L-malic, tartaric, citric, acetic, propionic and butyric acids. In contrast to such studies, we observed low-molecular weight compounds associated with Cd that could be either of organic or inorganic nature, including compounds like formic acid, propynoic acid, pyrazole, imidazole, alcohols, sulfurous acid or chloric acid. Plants release inorganic compounds into soil environment such as bicarbonates, hydroxides and ions (Ehrenfeld et al., 2005; Hartmann et al., 2009). None of them match our potential targets. Equally, a contribution by microbes to the observed metabolome needs to be recognized. Microbes when exposed to heavy metals mitigate against their toxic effects mainly via efflux systems, which pumps metals out of the cell, but also release of specific metal-binding chelators (Haferburg and Kothe, 2007; Gadd, 2010; Ahemad and Kibret, 2014). However, these microbial chelators were not noted in our metabolomics study. Another possibility is that the inorganic acids were associated with non-biological events and only coincided with heavy metals. Metals and inorganic acids form salts, which upon various environmental conditions might precipitate and become trapped inside a porous structure of soil particles (Gadd, 2010). Potentially they could have been released during the metabolite extraction procedure, thus resulting in the presence of specific, inorganic ions in the HPLC-MS profiles.

An interesting effect was observed with regard to bioavailable fraction of Zn. In most of the analyses performed in our work, such as PLFA, BIOLOG-CLPP or HPLC-MS, the effects of bioavailable Zn were highly variable and sometimes opposite compared to those seen with the total Zn fraction and other metals, like Cd and Cu. This may reflect that Zn is an essential metal and in low concentrations acts as a stimulant for the microbial communities (Bruins et al., 2000; Gadd, 2010). The impact of bioavailable Zn on individual biological parameters was in line with SOM and water content, as one would expect a soluble fraction of heavy metals. However copper, besides being an essential metal, did not show that kind of behavior. Furthermore, concentrations of bioavailable Zn were up to three orders of magnitude higher, than concentrations of bioavailable Cu. This distinctive effect could arise from tested microbial communities being derived from highly polluted, post-industrial areas associated with Zn processing/disposal and hence were subjected to prolonged environmental stress (40+ years) which could be linked to microbial adaptation.

Studies of microbial diversity in soil are difficult and involve a series of methodological challenges, including inability to culture vast majority of microorganisms present in the soil (Kirk et al., 2004; Alain and Querellou, 2009; Rastogi et al., 2010).

The results obtained through BIOLOG-CLPP are biased toward fast-growing and most active microorganisms, overshadowing viable, but slow-growing bacteria present in soil. However, this method allows for the comparison of functional biodiversity of tested microbial communities, enabling for calculating biodiversity indices and analysis through multivariate statistics (Kirk et al., 2004).

PLFA analysis provides quantitative estimates on viable microbial cells. This is due to the fact, that phospholipids are present in all living cells but are rapidly turned over after the cell's death (Fang et al., 2000). Unlike plate microbial counts or BIOLOG-CLPP, the PLFA technique is culture-independent and all extractable PLFAs are analyzed. However, the results obtained with this method depend on the PLFA extraction efficiency and many environmental factors (Macnaughton et al., 1997; Fang et al., 2000; Kirk et al., 2004; Wu et al., 2010).

In that matter, the PCR-based molecular methods are more robust and allow for significantly higher resolution, as high as individual species and microbial strains. 16S rDNA-targeted PCR-DGGE is reliable, reproducible, fast and inexpensive. Multiple samples can be analyzed in one run, allowing for simultaneous profile comparison, corresponding to microbial community structure. The major shortcomings of PCR-DGGE method are related to PCR itself, which include bias toward certain, abundant groups of microorganisms. The DNA isolation efficiency may vary depending on the method employed. In addition, depending on the DNA fragment mobility, one band may represent multiple species or sequences from the same species may result in more than one band (Kirk et al., 2004).

In our study, a combined use of culture-based and culture-independent approaches was aimed toward a thorough understanding of links between environmental factors and microbial traits, such as microbial counts as well as structural, functional and genetic diversity of the microbial communities in analyzed soils. Alternatively, the next-generation sequencing (NGS) methods could be implemented, allowing for deep sequencing, while mitigating many of the shortcomings of culture-dependent and traditional culture-independent approaches. Although the NGS approach allows for very fine resolution, only dominant OTUs are commonly analyzed and discussed (Teeling and Glöckner, 2012; Orgiazzi et al., 2015; Johnston-Monje et al., 2016).

## Conclusions

The difficulty in defining the key players in any environmental situation is well known due to the complexity of the various biotic and abiotic factors (Berg and Smalla, 2009). Our study shows, that despite the use of multiple assays, we could not define a single major parameter; assuming that they exist. However, we have made unequivocal conclusions regarding the significance of plant species, sampling site or heavy metals on the structure and function of rhizosphere microbial populations. Microbial community structure needs not correspond to community function because various microbial groups overlap in their functional potential. This process, called functional redundancy, allows microbial communities to maintain functional balance even when significant switches in the individual dominant species or genera occur (Nannipieri et al., 2003; Stefanowicz et al., 2008; McGuire and Treseder, 2010). Therefore the shifts in microbial CLPPs associated with heavy metal presence, as demonstrated in this work, are of great environmental significance and need to be explored in the future using now well-established microbiomic approaches.

## Data availability statement

Datasets are available on request: The raw data supporting the conclusions of this manuscript will be made available by the authors, without undue reservation, to any qualified researcher.

## Author contributions

The study was designed by SB. Most of the laboratory procedures, data analysis and manuscript writing was carried out by SB. ZP-S supervised the work. MC carried out the PCR-DGGE and helped with data analysis. LM and MB provided crucial help with HPLC-MS data processing and metabolomics. ZP-S, MC, and LM also contributed to manuscript writing.

### Conflict of interest statement

The authors declare that the research was conducted in the absence of any commercial or financial relationships that could be construed as a potential conflict of interest.
